# Retained Lumbar Drain Tip Leading to Intrathecal Hematoma: A Case Report on Perioperative Risks and Management

**DOI:** 10.7759/cureus.95169

**Published:** 2025-10-22

**Authors:** Seung J Lee, Kanchana Gattu, Joshua W Sappenfield

**Affiliations:** 1 Anesthesiology, University of Maryland School of Medicine, Baltimore, USA; 2 Anesthesiology, University of Maryland Medical Center, Baltimore, USA; 3 Anesthesiology, University of Florida, Gainesville, USA

**Keywords:** intrathecal catheter retention, intrathecal hematoma, lumbar drain complications, perioperative anticoagulation, thoracoabdominal aortic aneurysm (taaa)

## Abstract

Intrathecal catheters, commonly used for cerebrospinal fluid (CSF) drainage during thoracoabdominal aortic aneurysm (TAAA) repair, play a critical role in protecting the spinal cord and reducing the risk of paraplegia. However, their use is associated with significant complications, including infection, catheter retention, and hematoma formation, particularly in anticoagulated patients. We present the case of a 79-year-old woman who developed an intrathecal hematoma following lumbar drain removal, complicated by a retained catheter tip and perioperative anticoagulation therapy. Despite initial neurological improvement after lumbar drain placement, her condition deteriorated postoperatively, resulting in progressive neurological deficits and, ultimately, death. This case highlights the importance of meticulous technique in lumbar drain management, careful anticoagulation monitoring, and heightened vigilance for complications requiring prompt recognition and intervention.

## Introduction

Intrathecal catheters for cerebrospinal fluid (CSF) drainage, also known as lumbar drains, are routinely used during thoracoabdominal aortic aneurysm (TAAA) repairs to provide spinal cord protection and decrease the risk of paraplegia. In patients undergoing extent I or II TAAA repair, the incidence of paraplegia or paraparesis without CSF drainage has been reported as high as 13%, compared with 2.6% in those managed with prophylactic drainage, highlighting its protective effect on spinal cord perfusion [1]. Despite these benefits, intrathecal catheters are not without risk and are associated with several complications, including infection, intracranial hypotension, retained catheter fragments, and hemorrhagic events [2-4].

Like other neuraxial techniques, such as epidural and spinal anesthesia, lumbar drain placement and removal also carry a risk of spinal hematoma, which can lead to significant morbidity, including permanent neurological deficits and the need for urgent surgical intervention. The overall incidence of spinal epidural hematoma after neuraxial anesthesia in the general population is estimated at between one in 150,000 and one in 190,000, though rates appear higher in patients undergoing aortic surgery with lumbar drains [5]. In one series of 65 patients undergoing TAAA repair, 62 (95%) received a preoperative lumbar drain, and two (3.2%) developed intraspinal hemorrhagic complications [6]. The use of anticoagulant therapy, platelet dysfunction, and coagulopathy are recognized as major risk factors for hematoma formation. 

In this report, we describe an elderly woman who developed an intrathecal hematoma associated with a retained lumbar drain fragment following removal. Her course was complicated by neurologic decline and eventual death, although a direct causal relationship is difficult to establish given her significant comorbidities.

## Case presentation

A 79-year-old woman was transferred to the Medical Center for management of an aortic arch aneurysm and a large saccular thoracic aortic aneurysm. Her past medical history was significant for chronic obstructive pulmonary disease, pulmonary hypertension, chronic kidney disease, blindness, and dementia. She underwent thoracic endovascular aortic repair (TEVAR) with an aortic arch debranching procedure under general anesthesia, which proceeded without intraoperative complications.

On postoperative day (POD) 1, the patient developed acute paraplegia and was unable to move her lower extremities. The cardiac anesthesia team was emergently consulted, and a lumbar CSF drain was placed with some technical difficulty. Following drain placement, the patient regained partial motor function in her legs.

**Figure 1:**
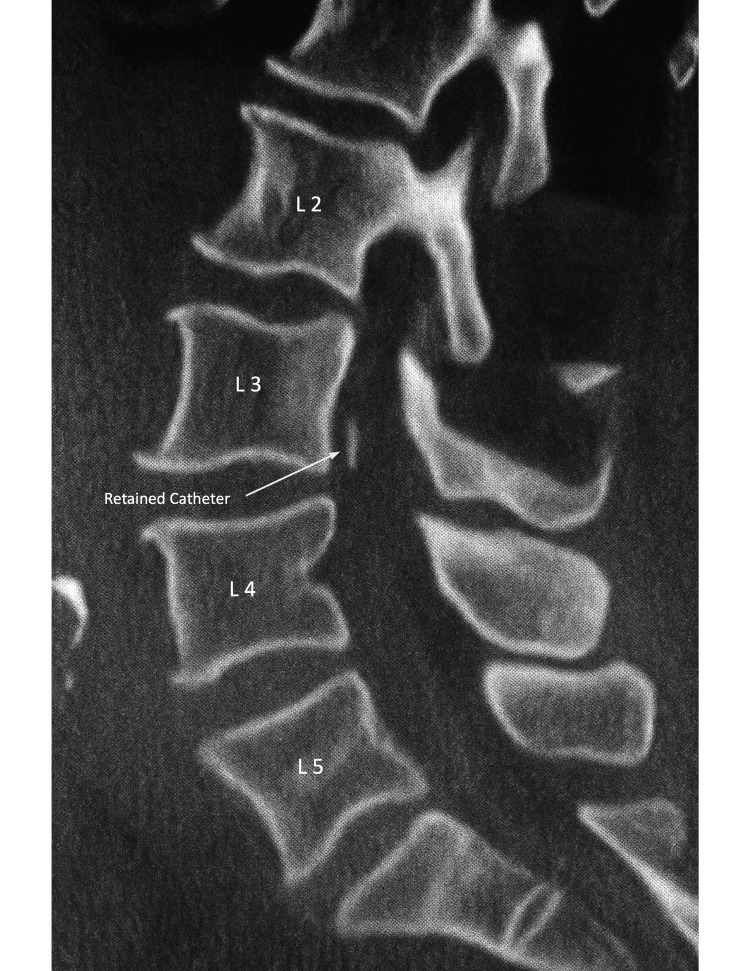
Lumbar spine CT Sagittal (lateral) view. Vertebral levels are labeled for orientation. The arrow indicates the retained catheter fragment within the intrathecal space.

**Figure 2:**
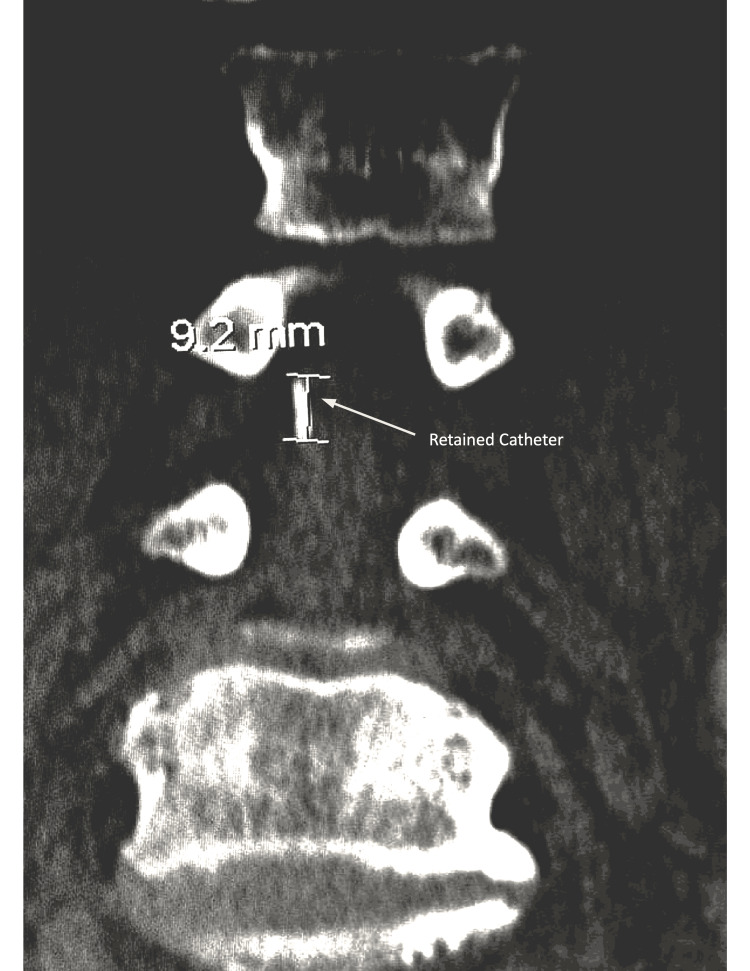
Lumbar spine CT Coronal view. The arrow indicates the retained catheter fragment.

Over the following days, her course was complicated by deep venous thrombosis of the right innominate vein and new-onset atrial fibrillation. Intravenous heparin therapy was started but discontinued when the CSF drainage became blood-tinged. On POD 4, per the institutional guidelines, the lumbar drain was removed after confirmation of a normalized activated partial thromboplastin time (aPTT). On inspection, the distal catheter tip was found to be missing. Lumbar CT demonstrated an intrathecal hematoma extending from L2 to the sacrum and a retained catheter fragment (Figures 1, 2). The neurosurgery service was consulted, and given the patient’s comorbidities and overall frailty, the decision was made to defer surgical intervention and pursue close neurologic monitoring.

On POD 5, the patient was restarted on subcutaneous heparin 5,000 units, and by 06:20 AM, her aPTT was >212 seconds, prompting discontinuation of anticoagulation. On POD 6, a superior vena cava filter was placed. MRI for further characterization of the intrathecal hematoma was attempted but not tolerated. 

By POD 8, the patient developed a right-sided pneumothorax, managed with supplemental oxygen and chest-tube placement. She continued to experience progressive neurologic decline, including complete loss of movement in the left lower extremity. She remained in the intensive care unit with ongoing clinical deterioration. Despite supportive management, her condition continued to decline, and she died on POD 24. Details of her clinical course are summarized in Table 1.

**Table 1:** Clinical course timeline TEVAR: thoracic endovascular aortic repair; DVT: deep vein thrombosis; IV: intravenous; CSF: cerebrospinal fluid; CT: computed tomography; aPTT: activated partial thromboplastin time; MRI: magnetic resonance imaging; LLE: left lower extremity; ICU: intensive care unit

Postoperative Day	Event / Clinical Course
0	TEVAR with aortic arch debranching; no anesthetic complications.
1	Acute paraplegia; emergent lumbar drain placed with difficulty → partial motor recovery.
2–3	New atrial fibrillation and right innominate vein DVT; started IV heparin → stopped when CSF became blood-tinged.
4	Drain removed; distal tip missing. CT: intrathecal hematoma (L2–sacrum) with retained catheter fragment. Neurosurgery recommended observation.
5	Subcutaneous heparin 5,000 U restarted; aPTT > 212 → anticoagulation stopped.
6	Superior vena cava filter placed; MRI attempt unsuccessful.
8	Right pneumothorax; worsening neurologic deficit (complete LLE paralysis).
9–23	Persistent ICU course with progressive deterioration.
24	Death.

## Discussion

Permanent neurological deficits after TAAA repair remain a serious concern, and many advocate for perioperative lumbar drainage to improve spinal cord perfusion pressure. When delayed spinal ischemia occurs, neurological deficits may sometimes be reversed if CSF drainage is initiated promptly after recognition of symptoms [1]. However, lumbar drainage is not without risk. Minor complications such as headache, nausea, and vomiting occur in up to 60% of patients [2]. Major complications, including infection, intracranial hypotension, retained catheter fragments, and spinal or epidural hematoma, are less frequent but potentially catastrophic [3,4]. In a 162-patient thoracic aortic series with lumbar drains, the overall mortality was 14.1%. Immediate permanent paraplegia occurred in 2.5% of patients, and delayed-onset paraplegia occurred in 9.3%, of whom 73% improved after lumbar CSF drainage with arterial pressure augmentation. The overall permanent paraplegia rate was 4.9%, and mortality among those with permanent paraplegia was 63%. Retained catheter fragments occurred in approximately 1.8% [7]. In our case, the exact timing of the intrathecal hematoma could not be determined; it may have occurred during the technically challenging placement or at the time of catheter removal. The patient’s need for anticoagulation in the setting of deep venous thrombosis and atrial fibrillation likely increased her risk for bleeding, consistent with previous studies identifying anticoagulation as a major contributor to neuraxial hematoma [3]. This combination of procedural difficulty, retained catheter fragment, and systemic anticoagulation culminated in a fatal outcome. This case emphasizes the delicate balance between the benefits and risks of lumbar CSF drainage. Meticulous attention to technique, inspection of catheter integrity upon removal, and immediate recognition of neurological deterioration are essential. Institutions should consider standardized protocols for drain management, particularly in patients who require perioperative anticoagulation.

## Conclusions

Perioperative lumbar drainage remains a critical tool for preventing spinal cord ischemia during TAAA repair; however, its use carries significant risks. This case highlights the potential for catastrophic complications, including retained catheter fragments and hematoma formation, particularly in anticoagulated patients. A structured approach to drain placement and removal, combined with vigilant neurological monitoring and individualized anticoagulation management, is essential to minimize morbidity and improve patient outcomes. Further research is warranted to refine best practices in lumbar drain management and to mitigate these associated risks.
